# Long‐term complete remission and peripheral biomarkers in Hodgkin lymphoma patients after decitabine‐plus‐camrelizumab epi‐immunotherapy and treatment cessation

**DOI:** 10.1002/mco2.428

**Published:** 2023-11-22

**Authors:** Chunmeng Wang, Yuting Pan, Yang Liu, Bing Guo, Jinhong Shi, Guanghua Rong, Zhipeng Guo, Zhifang Li, Qingming Yang, Jing Nie, Weidong Han

**Affiliations:** ^1^ The Second School of Clinical Medicine Southern Medical University Guangzhou China; ^2^ Department of Bio‐therapeutic the First Medical Centre, Chinese PLA General Hospital Beijing China; ^3^ Department of Bio‐therapeutic the Fifth Medical Centre, Chinese PLA General Hospital Beijing China; ^4^ Chinese People's Liberation Army Medical School Chinese PLA General Hospital Beijing China; ^5^ Changping Laboratory Beijing China

**Keywords:** biomarkers, classical Hodgkin lymphoma, decitabine‐plus‐camrelizumab, long‐term remission, relapse‐free survival, treatment discontinuation

## Abstract

Patients with relapsed/refractory classical Hodgkin lymphoma (cHL) achieve complete response (CR) after decitabine‐plus‐camrelizumab therapy, while long‐term outcome especially after treatment discontinuation remains unclear. We present a retrospective analysis of 87 relapsed/refractory cHL patients who acquired CR after decitabine‐plus‐camrelizumab. Patients were divided into two groups and received consolidation treatment every 3–4 or 6–12 weeks, and 1‐year of continuous CR was guaranteed for treatment cessation. At a median follow‐up of 5.3 years, the median relapse‐free survival (RFS) after achieving CR with decitabine‐plus‐camrelizumab therapy was 4.5 years, and patients underwent consolidation per 3–4 weeks might have longer RFS. The baseline percentage of peripheral central memory T cells was not associated with RFS, while patients with higher pretreatment serum levels of interleukin‐6 (IL‐6) and lactate dehydrogenase (LDH) had significantly shorter RFS and increased risk for disease recurrence. Fifty‐seven patients completed and discontinued decitabine‐plus‐camrelizumab, and their median RFS had not been reached. The 2‐year RFS rate after treatment cessation was 78% (95% CI, 67–90%). Patients in the high‐risk subgroup with higher pretreatment IL‐6 and LDH levels showed poor treatment‐free remission. Moreover, decitabine‐plus‐camrelizumab therapy was safe and cost‐effective. In conclusion, patients who obtained CR with decitabine‐plus‐camrelizumab and received consolidation per 3–4 weeks can achieve long‐term remission after treatment discontinuation.

## Graphical abstract

Patients with relapsed/refractory cHL who achieved CR with decitabine‐plus‐camrelizumab therapy were included, they had consolidation therapy (per 3–4 or 6–12 weeks), and those having 1‐year of continuous CR could discontinue treatment. The clinical benefits and serum biomarkers were investigated. Patients acquired satisfactory relapse‐free survival after decitabine‐plus‐camrelizumab cessation, especially low‐risk patients who had lower pretreatment serum levels of IL‐6 and LDH.

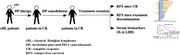

## 1 INTRODUCTION

Classic Hodgkin lymphoma (cHL) is a curable disease diagnosed at early stage, and approximately 10−30% of patients experience relapse or refractory disease. The standard of care for relapsed/refractory cHL includes high‐dose salvage chemotherapy and autologous stem cell transplantation (ASCT). However, up to 50% of patients will relapse after ASCT.^1^ As post‐ASCT maintenance, brentuximab vedotin (BV) and/or programmed death 1 (PD‐1) blockade therapy were effective with improved progression‐free survival (PFS).^2^, ^3^, ^4^

A proportion of patients with cHL were ineligible for ASCT owing to chemorefractory disease, age, or comorbidities. Among patients ineligible for ASCT or relapsing after ASCT, anti‐PD‐1 therapy resulted in objective response rate of 69−87% with 20−30% of cHL patients achieving complete remission.^5^, ^6^, ^7^, ^8^ To further increase complete response (CR) rate, anti‐PD‐1 antibody plus X (chemotherapy, other immune checkpoint inhibitors, epigenetic‐drugs or chimeric antigen T‐cells therapy) have been explored in patients with relapsed/refractory cHL.^9^, ^10^, ^11^, ^12^ We previously reported that among cHL patients (most were transplant‐ineligible or after ASCT), the combination of DNA demethylating agent decitabine and anti‐PD‐1 camrelizumab obtained prominently improved CR rate of 79% and prolonged PFS of 35 months in anti‐PD‐1‐naïve patients.^9^, ^13^ For patients relapsed/progressed after anti‐PD‐1 monotherapy, 50−60% of patients benefited from decitabine‐plus‐camrelizumab, including 30% of patients achieving CR, with median PFS of 20 months.^14^ In 2020, decitabine‐plus‐camrelizumab therapy became the guideline of relapsed/refractory cHL treatment in China.

PD‐1 blockade therapy displayed outstanding clinical response in cHL, and the long‐term remission durability of PD‐1 inhibitor in cHL patients who acquired CR was an important issue. A number of patients achieving CR after anti‐PD‐1‐based therapy proceed to subsequent allogeneic hematopoietic cell transplantation (alloHCT) and achieved a response, but significant immune‐related toxicity in the pre‐alloHCT setting could not be ignored.^15^, ^16^ Thus, it is valuable to clarify the extended antitumor response (during administration and cessation) in patients with relapsed/refractory cHL who achieved CR after anti‐PD‐1‐containing therapy and did not undergo ASCT or alloHCT. Moreover, investigating peripheral biomarkers to predict cHL relapses would be of great interest to the clinical practice.

To explore the above questions, we assembled and analyzed patients with relapsed/refractory cHL in our center that achieved CR after decitabine‐plus‐anti‐PD‐1 therapy. With abundant cases in CR and long‐term follow‐up, we expect to reveal the curable feasibility and biomarkers of anti‐PD‐1‐containing therapy in cHL patients who do not choose ASCT or previously treated with ASCT.

## 2 RESULTS

### 2.1 Patients

**FIGURE 1:**
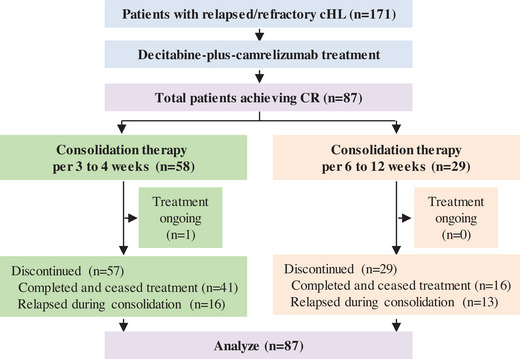
Study profile.

From January 2017 to September 2021, 171 patients with relapsed/refractory cHL from our clinical trial (NCT02961101 and NCT03250962) and from real world data who received decitabine‐plus‐camrelizumab therapy at our center were enrolled. At data cutoff on September 21, 2023, 87 patients acquired CR after decitabine‐plus‐camrelizumab therapy. After confirmed CR, patients received decitabine‐plus‐camrelizumab consolidation per 3–4 weeks or 6–12 weeks, and discontinued treatment after 1‐year of continued CR. The study profile and baseline characteristics for all patients in CR are summarized in Figure 1 and Table 1. There was no significant difference in sex, age, disease stage, tumor burden, and previous therapies between the two consolidation treatment groups (Table S1). Median age was 29 years (range, 12−73), and 52% of patients had stage IV disease at diagnosis. These patients were heavily pretreated with 66% having received three or more previous treatment regimens and 59% experiencing more than 10 cycles of chemotherapy. Fifty‐two patients (60%) had primary refractory disease that never obtained CR or relapsed within 3 months of the frontline therapy. Twenty‐four patients (28%) had undergone anti‐PD‐1 or anti‐PD‐L1 antibody (nivolumab, pembrolizumab, camrelizumab, or others) previously, and 24 patients (28%) received ASCT previously.

**TABLE 1:** Baseline characteristics of patients who achieved CR after decitabine‐plus‐camrelizumab therapy.

Characteristics	All patients (*n* = 87)	Consolidation per 3–4 weeks (*n* = 58)	Consolidation per 6–12 weeks (*n* = 29)
Median age, years (range)	29 (12–73)	31 (13–73)	27 (12–58)
Male sex, no. (%)	53 (61%)	34 (59%)	19 (66%)
Disease stage at diagnosis, no. (%)			
II	24 (28%)	16 (28%)	8 (28%)
III	18 (21%)	10 (17%)	8 (28%)
IV	45 (52%)	32 (55%)	13 (45%)
Tumor burden ≥20 cm2 at enrollment, no. (%)	36 (41%)	21 (36%)	15 (52%)
Lines of previous systemic therapy			
Median (range)	3 (1–14)	3 (1–14)	3 (1–11)
≥3 lines, no. (%)	57 (66%)	37 (64%)	20 (69%)
Cycles of previous chemotherapy			
Median (range)	11 (4–36)	11 (4–36)	10 (4–30)
≥10, no. (%)	51 (59%)	34 (59%)	17 (59%)
Primary refractory disease, no. (%)	52 (60%)	35 (60%)	17 (59%)
Previous ASCT, no. (%)	24 (28%)	15 (26%)	9 (31%)
Previous anti‐PD‐1/PD‐L1, no. (%)	24 (28%)	20 (34%)	4 (14%)

### 2.2 Relapse‐free survival

**FIGURE 2:**
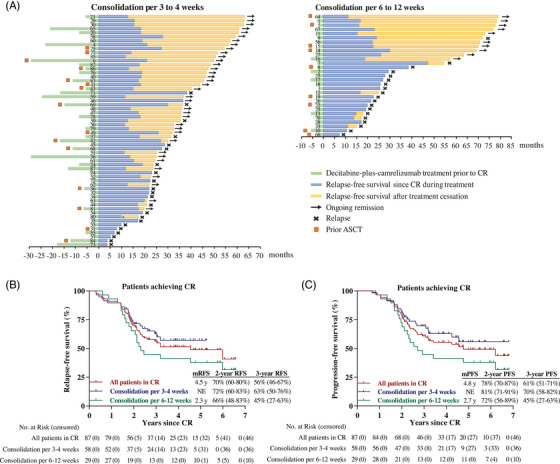
Relapse‐free survival. (A) Complete response duration in patients during treatment and after treatment cessation, separately by the two consolidation regimens. The length of green bars shows the time from first dosing until patients evaluated as CR with decitabine‐plus‐camrelizumab treatment. The length of blue bars shows the time from first CR until relapse or accomplished and discontinued decitabine‐plus‐camrelizumab treatment. Yellow bars indicate treatment‐free period, and the length of the bars shows the time from decitabine‐plus‐camrelizumab cessation until disease relapse or data cutoff date. Patients who had previous ASCT therapy were shown by orange squares. ^*^, this patient discontinued treatment by himself before continued CR reaching one year. (B and C) Kaplan–Meier survival curves of relapse‐free survival (RFS) since CR (B) and progression‐free survival (PFS, C). Curves for the overall patients (red line) and patients underwent consolidation per 3–4 weeks (blue line) or 6–12 weeks (green line) are shown. The median RFS (PFS), 2‐year and 3‐year RFS (PFS) rates (95% CI) are shown. Plus signs indicate censored data.

As of the cutoff date, median follow‐up from the initiation of therapy was 5.3 years (range, 1.9–6.7 years) and median follow‐up from patients in CR was 4.9 years (range, 1.7–6.6 years). Fifty‐seven patients (66%) completed treatment after having more than 1‐year of sustained CR and ceased treatment, including 41 patients undergoing consolidation every 3–4 weeks and 16 patients undergoing consolidation every 6–12 weeks. Twenty‐nine patients (33%) discontinued treatment due to disease relapse during consolidation therapy. Outcome for all patients during decitabine‐plus‐camrelizumab therapy and treatment cessation was summarized in Figure 2A.

The median relapse‐free survival (RFS) after decitabine‐plus‐camrelizumab in CR patients was 4.5 years and 2‐year RFS rate was 70% (95% CI, 60−80%) (Figure 2B). The median PFS in CR patients was 4.8 years with 2‐year PFS rate of 78% (95% CI, 70−87%) (Figure 2C). Patients undergoing consolidation per 3–4 weeks probably had longer RFS and PFS compared with those on consolidation per 6–12 weeks, while not statistically significant by long‐rank (Mantel–Cox) test (*p* = 0.129 for RFS, and *p* = 0.081 for PFS). The 3‐year RFS and PFS rates were 63% (95% CI, 50−76%) and 70% (95% CI, 58−82%) for patients who received consolidation every 3 to 4 weeks.

### 2.3 Risk factors for relapse‐free survival of cHL patients

**FIGURE 3:**
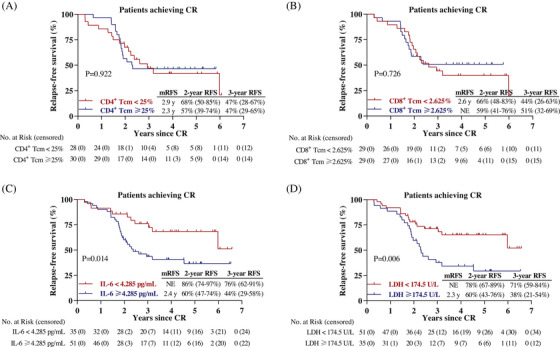
Predictive value of pretreatment serum IL‐6 and LDH. (A and B) Kaplan–Meier survival curves of relapse‐free survival in 58 patients stratified according to circulating CD4^+^ T_cm_ frequency (≥25 vs. <25%, A), and CD8^+^ T_cm_ frequency (≥2.625 vs. <2.625%, B). The median of CD4^+^ T_cm_ frequency and CD8^+^ T_cm_ frequency among the 58 patients were 25 and 2.625%, respectively. (C and D) Kaplan–Meier survival curves of relapse‐free survival in 86 patients stratified according to IL‐6 level (≥4.285 vs. <4.285 pg/mL, C), and LDH level (≥174.5 vs. <174.5 U/L, D). The cut‐off value of IL‐6 and LDH were clarified by ROC curves.

We previously reported that decitabine‐plus‐camrelizumab therapy resulted in incremental increase of circulating CCR7^+^CD45RA^−^ central memory T (T_cm_) cells in patient with relapsed/refractory cHL, especially those who acquired CR.^13^, ^14^ Among the 58 patients in this study who tested the peripheral T cell phenotype, there was no significant difference in RFS curves between the two groups, divided by the median percentage of T_cm_ cells, indicating that the baseline circulating T_cm_ frequency was not associated with CR durability after decitabine‐plus‐camrelizumab (Figures 3A and 3B).

We next explored the relationship between RFS and hematological biomarkers that were routinely tested. Based on the receiver operating characteristic (ROC) analysis in 86 patients, serum interleukin‐6 (IL‐6) and lactate dehydrogenase (LDH) may be potential prognostic biomarkers, while other variables, such as neutrophil‐to‐lymphocyte ratio (NLR), platelet–lymphocyte ratio (PLR), lymphocyte‐to‐monocyte ratio (LMR), and prognostic nutritional index (PNI) did not show marked likelihood ratio for area under the ROC curve (AUC). In term of IL‐6, AUCs for 2‐year, 3‐year, 4‐year, and 5‐year RFS were 0.654 (*p* = 0.025), 0.650 (*p* = 0.019), 0.629 (*p* = 0.040), and 0.653 (*p* = 0.023), respectively (Figure S1). The best cutoff value of IL‐6 and LDH were 4.285 pg/mL and 174.5 U/L. Univariate Cox regression analysis identified elevated serum levels of IL‐6 (HR = 2.350, *p* = 0.017) and LDH (HR = 2.343, *p* = 0.008) as possible prognostic indicators of RFS in cHL patients (Table S2).

Among the 86 cHL patients, the Kaplan–Meier curves elucidated that cHL patients with relatively lower IL‐6 level (<4.285 pg/mL), or lower LDH level (<174.5 U/L) had better RFS than those with higher IL‐6 level (≥4.285 pg/mL, *p* = 0.014), or higher LDH level (≥174.5 U/L, *p* = 0.006) (Figures 3C and 3D).

### 2.4 RFS after treatment cessation and biomarker prediction

**FIGURE 4:**
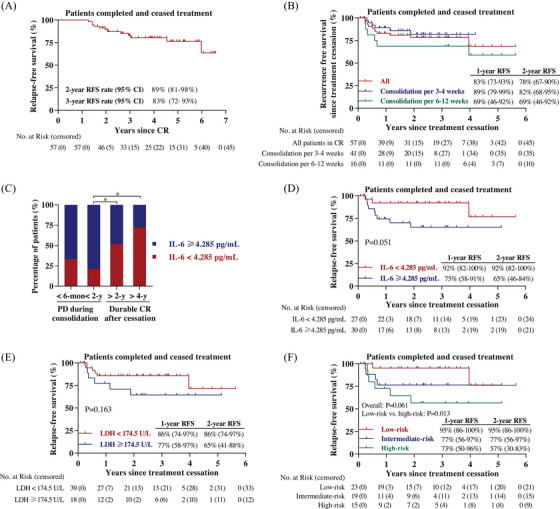
Relapse‐free survival since treatment cessation and serum biomarker analysis. (A) Kaplan–Meier survival curve of relapse‐free survival in patients who completed and discontinued treatment. (B) Kaplan–Meier survival curves of relapse‐free survival since discontinuation of decitabine‐plus‐camrelizumab therapy among all 57 patients (red line), and patients underwent consolidation per 3–4 weeks (blue line) or 6–12 weeks (green line). The RFS rates (95% CI) at 1 year and 2 years since treatment cessation are shown. (C) Distribution of patients with the higher or lower serum IL‐6 level among patients who had progressive disease during consolidation therapy within 6 months (*n* = 3), 1 year (*n* = 8), or had durable remission after treatment cessation for more than 2 years (*n* = 31), 4 years (*n* = 7). *p* Values are calculated using the *χ*2 test. CR, complete response. PD, progressive disease. (D and E) Kaplan–Meier survival curves of relapse‐free survival since treatment cessation stratified according to IL‐6 level (≥4.285 vs. <4.285 pg/mL, D), and LDH level (≥174.5 vs. <174.5 U/L, E). The 1‐year and 2‐year RFS rates (95% CI) since treatment cessation are shown. (F) Patients were defined as low‐risk, intermediate‐risk and high‐risk groups, who had both, either, or none of lower pretreatment IL‐6 (<4.285 pg/mL) and lower LDH (<174.5 U/L), respectively. The RFS rates (95% CI) at 1 year and 2 years since treatment cessation are shown. *, *p* < 0.05.

Patients could cease treatment after more than 1‐year of continuous CR. As data cutoff date, a total of 57 patients had completed and discontinued decitabine‐plus‐camrelizumab therapy; their median RFS was not obtained and RFS rates at 2 years and 3 years were 89% (95% CI, 81−98%) and 83% (95% CI, 72−93%), respectively (Figure 4A). With a median follow‐up of 3.0 years (range, 0.1–5.6 years) since treatment cessation, the 2‐year RFS rate since discontinuation was 78% (95% CI, 67−90%) and the median RFS was not reached (Figure 4B). Among the 39 patients who had more than 1 year of treatment‐free period, the vast majority of patients (92%) were still in CR. A total of 31 patients had been off therapy beyond 2 years and 30 patients remained disease free, including seven patients with long‐lasting remission for over 4 years. Although not statistically significant, consolidation treatment every 3–4 weeks showed higher 1‐year RFS rate since treatment cessation as opposed to 6–12‐week regimen (89 vs. 69%) (Figure 4B).

We further examined the relevance of baseline serum biomarker and clinical outcome. The proportion of patients with low IL‐6 level (<4.285 pg/mL) in the subgroup who undergone tumor relapse during consolidation therapy within 2 years was decreased compared with those who maintained long‐term CR after treatment cessation for over 2 years (Figure 4C). Moreover, improved RFS since treatment discontinuation was observed for patients with low level of serum IL‐6 or LDH (Figures 4D and 4E). To more precisely predict treatment‐free CR duration, patients who had both, either or none lower pretreatment IL‐6 and LDH levels were defined as low‐risk, intermediate‐risk, or high‐risk subgroups, respectively. Notably, treatment‐free RFS results were different among these subgroups (Figure 4F, overall *p* = 0.061, low‐risk vs. high‐risk *p* = 0.013).

### 2.5 Adverse events

During the long‐term follow‐up for patients with relapsed/refractory cHL who acquired CR after decitabine‐plus‐camrelizumab therapy, the most common treatment‐related adverse events (AEs) were reactive capillary endothelial proliferation (86.2% for consolidation every 3–4 weeks and 79.3% for consolidation every 6–12 weeks) and leukocytopenia (84.5% for consolidation every 3–4 weeks and 79.3% for consolidation every 6–12 weeks). Other common AEs among all patients were pyrexia (27.6%) and increased triglyceride (24.1%) (Table 2).

**TABLE 2:** Treatment‐ related adverse events (any grade ≥5%).

Characteristics	All patients (*n* = 87)		Consolidation per 3–4 weeks (*n* = 58)		Consolidation per 6–12 weeks (*n* = 29)	
	Any grade	Grade ≥ 3	Any grade	Grade ≥ 3	Any grade	Grade ≥ 3
Any adverse event	85 (97.7%)	22 (25.3%)	57 (98.3%)	15 (25.9%)	28 (96.6%)	7 (24.1%)
Reactive capillary endothelial proliferation	73 (83.9%)	0	50 (86.2%)	0	23 (79.3%)	0
Leukocytopenia	72 (82.8%)	22 (25.3%)	49 (84.5%)	15 (25.9%)	23 (79.3%)	7 (24.1%)
Pyrexia	24 (27.6%)	0	16 (27.6%)	0	8 (27.6%)	0
Increased triglyceride	21 (24.1%)	0	19 (32.8%)	0	2 (6.9%)	0
Increased transaminase	11 (12.6%)	0	8 (13.8%)	0	3 (10.3%)	0
Thrombocytopenia	11 (12.6%)	1 (1.1%)	9 (15.5%)	0	2 (6.9%)	1 (3.4%)
Pain in the lesion	11 (12.6%)	0	8 (13.8%)	0	3 (10.3%)	0
Increased TSH	7 (8.0%)	0	6 (10.3%)	0	1 (3.4%)	0
Myalgia	7 (8.0%)	0	3 (5.2%)	0	4 (13.8%)	0
Nausea	6 (6.9%)	0	4 (6.9%)	0	2 (6.9%)	0
Rash	6 (6.9%)	0	3 (5.2%)	0	3 (10.3%)	0
Diarrhea	6 (6.9%)	0	3 (5.2%)	0	3 (10.3%)	0
Fatigue	5 (5.7%)	0	4 (6.9%)	0	1 (3.4%)	0
Increased UA	5 (5.7%)	0	2 (3.4%)	0	3 (10.3%)	0

## 3 DISCUSSION

In this retrospective analysis with median follow‐up of 5.3 years, we reported the long‐term outcomes of 87 patients with relapsed/refractory cHL who acquired CR after decitabine‐plus‐camrelizumab epi‐immunotherapy and 57 patients therein discontinued treatment, the largest case of CR patients after anti‐PD‐1 therapy with long observation ever published to our knowledge. Our results showed that 56% of patients sustained remission at 3 years since achieving CR after decitabine‐plus‐camrelizumab and without undergoing stem cell transplantation. Patients could discontinue treatment if continuous CR was remained for more than 1 year, and consolidation therapy per 3–4 weeks was recommended. Strikingly, relatively lower level of circulating IL‐6 was associated with longer RFS and lower risk of relapse in cHL patients.

For patients with relapsed/refractory cHL, high‐dose chemotherapy and ASCT acts as the standard of care and offers long‐term remissions in half of patients.^1^ Among 115 cHL patients, the 5‐year PFS rates for all patients and patients in CR after ASCT was 53 and 69.2%, respectively.^17^ When PD‐1 blockade was used as consolidation after ASCT, 82% of patients remained response at 18 months.^2^ With BV as post‐ASCT consolidation, the 5‐year PFS rate reached 59%.^3^ Patients relapsing following ASCT had poor outcome with the reported OS at 5 years of 32%.^18^ In this present study, we demonstrated the durable antitumor responses for patients in CR after decitabine‐plus‐camrelizumab therapy and without receiving stem cell transplantation, especially undergoing consolidation every 3–4 weeks (2‐year and 3‐year PFS rates of 81 and 70%, respectively), which was comparable with outcome from patients undergoing ASCT plus post‐ASCT pembrolizumab maintenance (18‐month PFS of 82%).^2^ High procedure costs and treatment‐related AEs were two major concerns for refusing ASCT in China, the mean cost for conducting one ASCT was estimated as ¥200,000.^19^ Contrastingly, toxicities and economic burden for cHL patients who received decitabine‐plus‐anti‐PD‐1 therapy were reduced versus those undergoing ASCT. Additionally, it was more cost effective to use decitabine plus camrelizumab, a domestic anti‐PD‐1 antibody, for Chinese patients with relapsed/refractory cHL.

Immune checkpoint blockade therapy was reported to produce prolonged response after treatment discontinuation in patients with melanoma or non‐small cell lung cancer.^20^, ^21^ What happens when anti‐PD‐1 therapy is discontinued and the optimal consolidation schedule before cessation remain unclear in cHL. Manson et al.^22^ reported that eight (80%) among the 10 patients with relapsed/refractory cHL in CR after nivolumab sustained prolonged remission after a median follow‐up of 22.4 months from nivolumab cessation. Another retrospective study presented that 11 (48%) patients relapsed after treatment discontinuation among the 23 patients with relapsed/refractory cHL who acquired CR following nivolumab therapy, and nine patients relapsed within 18 months since treatment cessation.^23^ Our study included 57 patients who discontinued decitabine‐plus‐camrelizumab therapy, the largest number of sample size ever published, nearly 80% of patients therein remained remission after a median follow‐up of 3 years since treatment cessation. Therefore, decitabine‐plus‐camrelizumab therapy might be a potentially curative approach in a proportion of cHL patients, especially on 3–4‐week consolidation protocol, rather than only as a bridge to transplantation.

Refractory cHL, extranodal involvement, bulky or advanced‐stage disease have been identified as poor prognostic factors for patients undergoing ASCT.^3^, ^24^ Here, we observed that RFS duration was similar in patients with refractory or relapsed disease, with or without extranodal involvement, and regardless of the number of prior lines, chemotherapy regimens or prior ASCT. To search potential biomarkers for CR duration, we collected parameters that were routinely measured in the clinical practice of immunotherapy, including IL‐6 and so on. High serum IL‐6 level was predictive of poor efficacy and short PFS in patients with non‐small‐cell lung cancer, and decrease in IL‐6 was associated with anti‐PD‐1 responses.^25^, ^26^ Moreover, IL‐6 level was correlated to early disease progression in melanoma patients receiving anti‐PD‐1 therapy.^27^ To our knowledge, the prognostic variables in cHL treated with PD‐1 blockade therapy have not been clearly examined. In this study, we reported the significance of serum baseline IL‐6 for cHL patients to predict remission duration of anti‐PD‐1‐combined immunotherapy. Based on our results, cHL patients in the high‐risk group with elevated levels of IL‐6 and LDH might have a poor prognosis, and these patients were not recommended to discontinue treatment even if they achieved 1‐year of sustained CR.

Exploring mechanism of cHL relapse after immunotherapy is crucial. We did not detect significant RFS difference between patients with distinct baseline peripheral T_cm_ frequencies, and thus the characteristic of Hodgkin RS (HRS) malignant cells might be related to the relapse of cHL after decitabine‐plus‐camrelizumab therapy. Besides PD‐L1, other tumor prognostic biomarkers for PD‐1/PD‐L1 blockade therapy have been investigated.^28^ HRS cells expressed IL‐6, which exerted as autocrine or paracrine to promote tumor cell growth by triggering the activation of JAK/STAT3 signaling, which was essential for the development of cHL.^29^ IL‐6 could also activate the immunosuppressive regulatory T cells, myeloid derived suppressor cells and tumor‐associated macrophages.^30^, ^31^ In tumor‐bearing mice, blockade of IL‐6 promoted the infiltration of CD8^+^ T cells and IFN‐γ^+^CD4^+^ T cells and sensitized anti‐PD‐1 therapy.^32^, ^33^ Theoretically, elevated IL‐6 was likely to contribute to immune evasion and disease relapse after anti‐PD‐1 therapy; investigation of single‐cell landscape of cHL is required and under way, in order to clarify the detailed mechanism underlying the effective antitumor response and resistance to epi‐immunotherapy in patients with relapsed/refractory cHL.

Our study has several limitations. This was a single‐center study and the AUC values of the potential indicators IL‐6 and LDH were not high enough. The prognostic biomarker study should be conducted in multicenters and greater number of patients. Moreover, the dynamic alteration of serum parameters should be evaluated, especially when cHL relapses.

In summary, our results show that patients with relapsed/refractory cHL who acquired CR after decitabine‐plus‐camrelizumab and underwent consolidation every 3–4 weeks had durable remission after treatment cessation. Pretreatment serum IL‐6 and LDH were promising prognostic indicators for RFS and high‐risk patients were not suggested to discontinue treatment.

## 4 MATERIALS AND METHODS

### 4.1 Patients

The patients included in this study were relapsed/refractory cHL patients who participated in our clinical trials (NCT02961101 and NCT03250962) or came from real world data in our center at Chinese PLA General Hospital. Eligible patients had diagnosis of relapsed/refractory cHL, were 12 years or older, with Eastern Cooperative Oncology Group performance‐status of 0 or 1, had adequate hematological, hepatic, and renal function, received at least eight courses of decitabine‐plus‐camrelizumab treatment and were evaluated as confirmed CR. The standard frontline therapy (ABVD or BECOOP) must be experienced. Participation was not restricted with regard to prior ASCT or BV. Key exclusion criteria included ASCT within 100 days, prior chemotherapy or other antitumor antibodies within 4 weeks, current autoimmune disease, any active infection, ≥ grade 3 organ toxicity because of prior immunotherapy, serious uncontrolled medical disorders, or a history of organ transplantation. All histological results were read by at least two pathologists. The clinical trial protocol was approved by the Institutional Review Board of the Chinese PLA General Hospital (S2016‐127‐01) and written informed consent was obtained.

### 4.2 Treatment

Decitabine was administered intravenous on days 1−5 (10 mg/d) and camrelizumab on day 8 (200 mg) every 3 weeks for the first eight cycles and per 3–4 weeks or 6–12 weeks during consolidation period. Tumor response was assessed at baseline, every two cycles by ultrasound or CT imaging, as well as by fluorine‐18 deoxyglucose PET/CT at baseline and every four cycles. CR assessment was confirmed by PET/CT with a Deauville score ≤ 3 considered a complete metabolic response, according to the Revised Response Criteria for Malignant Lymphoma.^34^ Patients with confirmed CR received decitabine‐plus‐camrelizumab consolidation therapy either per 3–4 weeks or 6–12 weeks until CR duration maintained for 1 year. As prespecified, patients in CR before January 1, 2019 received consolidation treatment every 6–12 weeks and if the PFS rate at 1.5 years was below 90% at that time, those CR patients evaluated after January 1, 2019 received consolidation every 3–4 weeks. 1‐year of continuous CR was guaranteed for treatment cessation and patients received no subsequent therapy until disease relapsed. During consolidation therapy, patients with appearance of any new PET positive lesions could be assessed temporarily as having indeterminate response (IR),^35^ and treatment should be continued until disease progression was confirmed by additional PET/CT.

### 4.3 Data collection

The absolute platelet count, absolute neutrophil count (ANC), absolute monocyte count (AMC), absolute lymphocyte count (ALC), serum albumin (ALB), C reactive protein (CRP), IL‐6, and LDH were collected from 86 cHL patients within 1 week prior to treatment start, and calculated the value of NLR, PLR, LMR, and PNI; these data from one patient was missing. NLR, PLR, LMR, and PNI were calculated using the following formula: ANC/ALC, platelet/ALC, ALC/AMC, and 1 × ALB (g/L) + 5 × ALC (10^9^/L), respectively.

### 4.4 Endpoints and statistical analysis

Patient baseline characteristics were described. The primary endpoint was RFS. RFS indicated the time from the first documented CR after decitabine‐plus‐camrelizumab to relapse or death, and RFS after treatment cessation was the period from treatment cessation to relapse or death. Patients who did not relapse at data cutoff date or lost to follow‐up were censored.

Survival curve was estimated using the Kaplan–Meier method, and the log‐rank test was used for between‐group comparisons. The best cut‐off value of IL‐6 and LDH were clarified by the ROC curves. Univariate analysis was performed using Cox proportional hazards model. All tests were two‐sided, and *p* value < 0.05 was considered significant. All statistical analyses were conducted using SPSS v26 and GraphPad Prism 9. Safety analyses were performed in all patients, and grade of severity was determined according to CTCAE version 5.0.

## AUTHOR CONTRIBUTIONS

C. W. and Y. P. collected and analyzed the data, and wrote the paper; Y. L., G. R., and Q. Y. guided the research; J. S., B. G., Z. G., and Z. L. collected the data; J. N. designed the study, analyzed the data, and wrote the paper; W. H. designed and conducted the study. All authors have read and approved the final manuscript.

## CONFLICT OF INTEREST STATEMENT

All authors declared no potential conflict of interest.

## ETHICS STATEMENT

The study was approved by the Ethics Committee of Chinese PLA General Hospital (S2016‐127‐01) and registered in ClinicalTrials.gov (NCT02961101 and NCT03250962), in accordance with the ethical principles of Good Clinical Practice and the Declaration of Helsinki. Written informed consent was obtained from all participants.

## Supporting information

Supporting Information

## Data Availability

The data supporting the findings of this study are available from the corresponding author upon reasonable request.
