# Supplementary material for: Long‐term complete remission and peripheral biomarkers in Hodgkin lymphoma patients after decitabine‐plus‐camrelizumab epi‐immunotherapy and treatment cessation
Source: MedComm (2020). 2023 Nov 22;4(6):e428. doi: 10.1002/mco2.428 (PMC10665599; doi:10.1002/mco2.428)
Supplement: Supplementary file 1 — Supporting Information [file MCO2-4-e428-s001.docx]

**Supplemental data**

**Long-term complete remission and peripheral biomarkers in Hodgkin lymphoma patients after decitabine-plus-camrelizumab epi-immunotherapy and treatment cessation**

Chunmeng Wang, Yuting Pan, Yang Liu, Bing Guo, Jinhong Shi, Guanghua Rong, Zhipeng Guo, Zhifang Li, Qingming Yang, Jing Nie, Weidong Han

**Contents**

Supplemental Figures 3

Figure S1. ROC curves for different peripheral markers. 3

Supplemental Tables 4

Table S1. Clinicopathological variables within subgroups. 4

Table S2. Univariate analysis of factors associated with RFS. 5

# Supplemental Figures

## Figure S1. ROC curves for different peripheral markers.


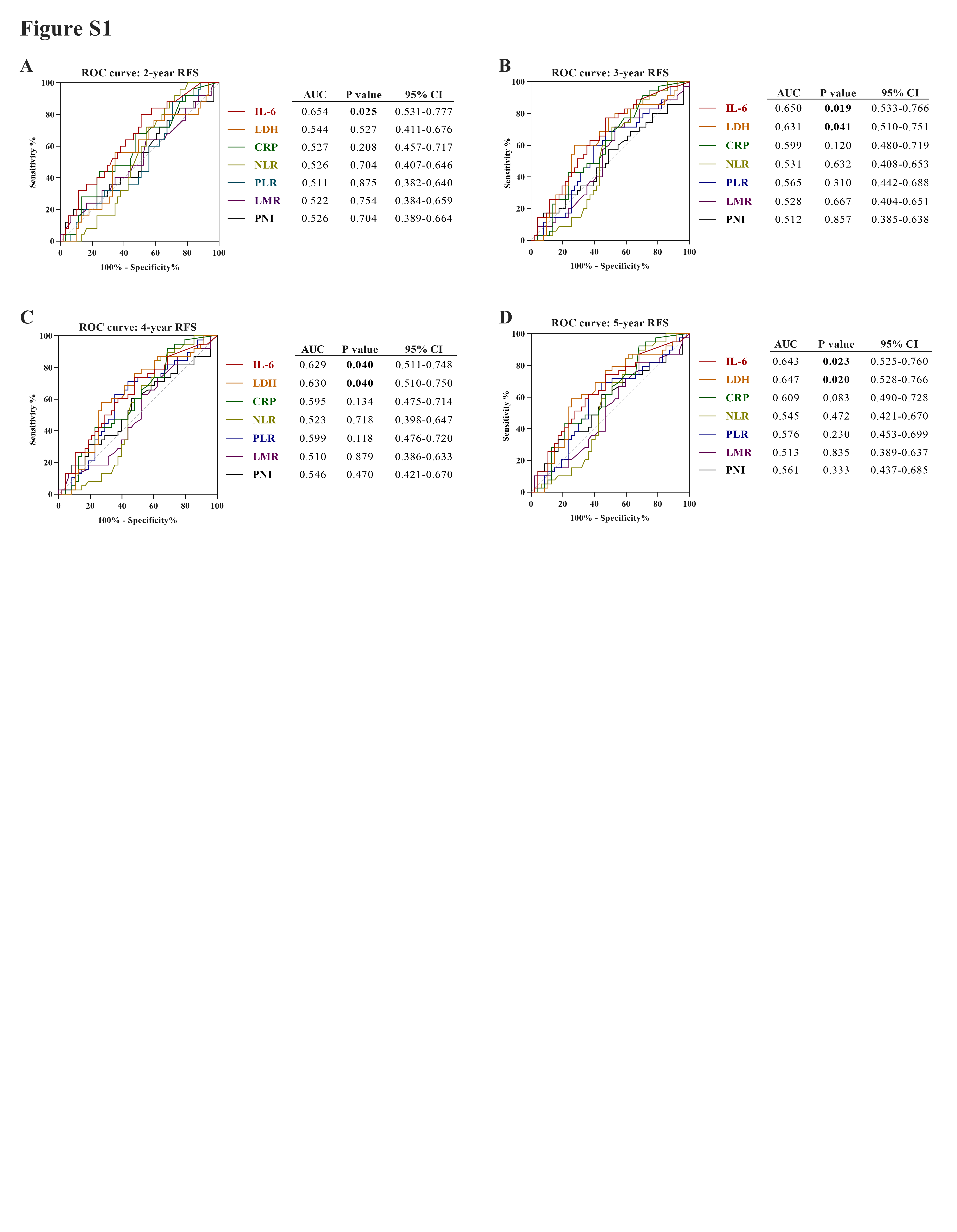


Receiver operator characteristic (ROC) curves to evaluate the predictability of RFS at 2-year (A), 3-year (B), 4-year (C), and 5-year (D). IL-6, Interleukin-6; LDH, lactate dehydrogenase; CRP, C-reactive protein; NLR, neutrophil-lymphocyte ratio; PLR, platelet-lymphocyte ratio; LMR, lymphocyte-to-monocyte ratio; PNI, prognostic nutritional index.

# Supplemental Tables

## Table S1. Clinicopathological variables within subgroups.

| **Characteristics** | **3 to 4-week consolidation (n=62)** | **6 to 12-week consolidation (n=34)** | **P value** |
| --- | --- | --- | --- |
| **Sex** |  |  |  |
| Male | 34 | 19 | 0.534 |
| Female | 24 | 10 |  |
| **Age (years)** |  |  |  |
| < 29 | 23 | 16 | 0.170 |
| ≥ 29 | 35 | 13 |  |
| **Stage** |  |  |  |
| II | 16 | 8 | 1.000 |
| III/IV | 42 | 21 |  |
| **Tumor burden (SPD)** |  |  |  |
| < 20 cm^2^ | 37 | 14 | 0.186 |
| ≥ 20 cm^2^ | 21 | 15 |  |
| **Previous lines of therapy** |  |  |  |
| < 3 | 21 | 9 | 0.632 |
| ≥ 3 | 37 | 20 |  |
| **Cycles of previous chemotherapy** |  |  |  |
| < 11 | 27 | 16 | 0.448 |
| ≥ 11 | 31 | 13 |  |
| **Primary refractory disease** |  |  |  |
| Yes | 35 | 17 | 0.877 |
| No | 23 | 12 |  |
| **Previous ASCT** |  |  |  |
| Yes | 15 | 9 | 0.611 |
| No | 43 | 20 |  |

SPD, sum of the products of the longest perpendicular diameters of tumors.

## Table S2. Univariate analysis of factors associated with RFS.

| **Patient Characteristics** | **Univariate analysis** | |
| --- | --- | --- |
|  | **HR (95% CI)** | **P-value** |
| IL-6 (≥ 4.285 vs. < 4.285 mg/L) | 2.350 (1.168-4.728) | **0.017** |
| LDH (≥ 174.5 vs. < 174.5 U/L) | 2.343 (1.248-4.400) | **0.008** |
| Sex (Male vs. Female) | 0.722 (0.384-1.354) | 0.310 |
| Age (≥ 29 vs. < 29 years) | 0.916 (0.485-1.731) | 0.787 |
| Stage at diagnosis (III/IV vs. II) | 1.845 (0.816-4.174) | 0.141 |
| Tumor burden (≥ 20 vs. < 20 cm^2^) | 1.281 (0.687-2.392) | 0.436 |
| Extranodal involvement (Yes vs. No) | 0.956 (0.501-1.826) | 0.892 |
| Primary refractory (Yes vs. No) | 0.652 (0.351-1.212) | 0.176 |
| Lines of prior therapy (≥ 3 vs. < 3) | 0.828 (0.431-1.591) | 0.571 |
| Cycles of prior chemotherapy (≥ 11 vs. < 11) | 1.259 (0.671-2.360) | 0.473 |
| Prior ASCT (Yes vs. No) | 0.817 (0.399-1.675) | 0.582 |
| Prior anti-PD-1/PD-L1 therapy (Yes vs. No) | 0.771 (0.364-1.632) | 0.496 |
| Consolidation therapy (6 to 12-week vs. 3 to 4-week) | 1.677 (0.891-3.156) | 0.109 |
